# Accounting for red blood cell accessibility reveals distinct invasion strategies in *Plasmodium falciparum* strains

**DOI:** 10.1371/journal.pcbi.1007702

**Published:** 2020-04-21

**Authors:** Francisco Y. Cai, Tiffany M. DeSimone, Elsa Hansen, Cameron V. Jennings, Amy K. Bei, Ambroise D. Ahouidi, Souleymane Mboup, Manoj T. Duraisingh, Caroline O. Buckee

**Affiliations:** 1 Center for Communicable Disease Dynamics, Department of Epidemiology, Harvard TH Chan School of Public Health, Boston, Massachusetts, United States of America; 2 Department of Immunology and Infectious Diseases, Harvard TH Chan School of Public Health, Boston, Massachusetts, United States of America; 3 Laboratory of Bacteriology and Virology, Le Dantec Hospital, Cheikh Anta Diop University, Dakar, Senegal; Emory University, UNITED STATES

## Abstract

The growth of the malaria parasite *Plasmodium falciparum* in human blood causes all the symptoms of malaria. To proliferate, non-motile parasites must have access to susceptible red blood cells, which they invade using pairs of parasite ligands and host receptors that define invasion pathways. Parasites can switch invasion pathways, and while this flexibility is thought to facilitate immune evasion, it may also reflect the heterogeneity of red blood cell surfaces within and between hosts. Host genetic background affects red blood cell structure, for example, and red blood cells also undergo dramatic changes in morphology and receptor density as they age. The *in vivo* consequences of both the accessibility of susceptible cells, and their heterogeneous susceptibility, remain unclear. Here, we measured invasion of laboratory strains of *P*. *falciparum* relying on distinct invasion pathways into red blood cells of different ages. We estimated invasion efficiency while accounting for red blood cell accessibility to parasites. This approach revealed different tradeoffs made by parasite strains between the fraction of cells they can invade and their invasion rate into them, and we distinguish “specialist” strains from “generalist” strains in this context. We developed a mathematical model to show that generalist strains would lead to higher peak parasitemias *in vivo* compared to specialist strains with similar overall proliferation rates. Thus, the ecology of red blood cells may play a key role in determining the rate of *P*. *falciparum* parasite proliferation and malaria virulence.

## Author summary

The growth of the malaria parasite Plasmodium falciparum in human blood causes all the symptoms of malaria. Parasites invade red blood cells using pathways defined by a parasite ligand and a corresponding host receptor. As red blood cells age, they undergo changes, affecting which pathways can be used by parasites for invasion. We studied laboratory parasites relying on distinct pathways and measured their ability to invade red blood cells of different ages. We found that parasites trade-off between the proportion of red blood cells they can invade and the efficiency of the invasion process. Using a mathematical model, we predict that these trade-offs affect parasite growth in vivo, with important consequences for virulence.

## Introduction

Proliferation of *Plasmodium falciparum* in human red blood cells underlies all clinical manifestations of malaria, and higher parasite densities in the blood are associated with more severe disease [1,2]. Parasite growth relies on the invasion of red blood cells, mediated by interactions between specific pairs of parasite ligands and host receptors, with each ligand-receptor pair defining a molecular invasion pathway [3]. Parasites can switch between invasion pathways via genetic and epigenetic mechanisms; these systems are thought to have evolved in the parasite to evade immune responses against particular ligands [4–7]. Redundant invasion pathways may also represent an adaptation to accommodate within- and between-host diversity in red blood cell structure and receptor composition [8]. While the importance of red blood cell genetic polymorphisms within human populations in determining parasite growth and disease severity has long been recognized [9–16], the substantial diversity of red blood cells within the host has received less attention. The factors that determine invasion pathway preferences *in vivo*, and their consequences for parasite growth and virulence, remain unclear.

Red blood cells are highly heterogeneous targets for invasion, exhibiting dramatic changes in both the composition and density of potential receptors over the course of their 4-month lifespan. For example, oxidative damage accumulated over this period results in the loss of 10–15% of the cell’s sialic acid (SA) content, reducing the number of glycophorin receptors that can be used by the parasite to enter cells [17–23]. Restrictions in receptor availability across differently aged red blood cells are likely to significantly impact the parasite’s *in vivo* proliferation. Indeed, differences in peak parasitemia and virulence between *P*. *falciparum* and *P*. *vivax* have been attributed to the restriction of *P*. *vivax* to invading reticulocytes, the youngest red blood cells [24,25]; by contrast, *P*. *falciparum* can invade a broader age range of cells, although it too exhibits a preference for younger cells [26]. The bloodstream represents a complex and changing ecology, particularly during chronic blood-stage infections in which the age distribution of red blood cells may shift over time, as well as in regions where super-infection is common, forcing parasites to compete for resources. As a result, flexible strategies for invasion may be key to enhancing parasite fitness within individual hosts.

The *P*. *falciparum* genome encodes at least 10 invasion ligands belonging to erythrocyte-binding like (EBL) and reticulocyte-binding like (RBL) protein families [19,27], each of which binds to a specific red blood cell receptor. Although not all host receptors have been identified, in general, EBL ligands rely on SA-based glycophorin receptors, whereas RBL ligands bind less well-defined receptors that include the recently identified Basigin and Complement Receptor 1 [20,28]. *In vitro*, different *P*. *falciparum* laboratory strains exhibit distinct preferences for particular invasion pathways [29–31]. This flexibility has also been observed in field isolates, with parasite lines from Kenya, Senegal, and the Gambia demonstrating diverse invasion pathway utilization [32–34]. Some parasites can switch at high frequency between alternative pathways [29]. The different members of the RBL and EBL ligands are not essential to invasion in all *P*. *falciparum* strains, with the PfRh5 parasite ligand being the exception [20,35], suggesting a functional redundancy in invasion strategies that provides flexibility in the face of immune responses and the heterogeneous ecology of red blood cell receptors.

The importance of different invasion pathways for infection dynamics and parasite growth *in vivo* is poorly understood. Metrics designed to measure the potential for parasite growth in the host using parasites cultured *in vitro* include the commonly measured parasite multiplication rate (PMR), which is analogous to the basic reproduction number (R_0_) in disease ecology, and the selectivity index (SI), which uses the ratio of observed-to-expected frequency of multiply invaded red blood cells to quantify the restriction of invasion to particular subsets of cells. However, both have exhibited inconsistent relationships with peak parasite densities and virulence *in vivo*; PMR calculated from the trajectory of controlled infections in patients with neurosyphilis was shown in the early 20^th^ century to be correlated with peak parasite densities [36]. Some studies of natural infections in Southeast Asia have also shown a correlation between PMR and disease severity, and an inverse correlation between SI and severity [24,37], but African studies have uncovered no such relationships [32,38].

We propose that variation in parasite proliferation *in vivo* among *P*. *falciparum* genotypes may reflect diverse ecological strategies–mediated by the use of different invasion pathways–in heterogeneous red blood cell populations. In other words, *P*. *falciparum* strains of distinct genotype may be specialized with respect to the ability to invade red blood cells of different age, just as different species of *Plasmodium* are. To investigate this, we measured the ability of laboratory-adapted strains of *P*. *falciparum* with known differences in invasion pathway utilization to enter red blood cells of different ages. We developed a method to jointly estimate the fraction of red blood cells available for invasion and the invasion rate into available red blood cells, to account for the fact that many cells may not be available for invasion simply because they are physically inaccessible to parasites. Our approach revealed different ecological strategies between parasite strains with respect to the age of the red blood cell that are not captured using standard methods. Using a dynamic model of parasite invasion and growth *in vivo*, we predict that these differences would lead to different infection dynamics and peak parasite densities. The complex ecology and dynamics of human blood is therefore an important but under-studied driver of malaria infection dynamics, providing the backdrop for variation in malaria parasite virulence.

## Results

### Accounting for red blood cell accessibility reveals variable age preferences between *P*. *falciparum* strains

To disentangle the implications of invasion pathway variation and the changing availability of host receptors as red blood cells age, we separated red blood cells into different age fractions (see Methods). We conducted invasion assays into these red blood cell subpopulations with four *P*. *falciparum* parasite laboratory strains (3D7, Dd2, HB3, and FCR3-SV) that use alternative invasion pathways, characterized by their dependence on SA-containing or chymotrypsin- or trypsin-sensitive receptors [19,29]. In addition, we analyzed the invasion of two strains, Dd2Nm and C2, which are isogenic to Dd2 and 3D7 respectively, with changes in expression of single invasion ligands (increased Rh4 expression in Dd2Nm) (39) and loss of Rh2b expression in C2 (40), causing them to shift in their relative dependence on SA for invasion. Invasion assays were plated at the same pre-invasion parasitemia, and the post-invasion parasitemia was measured. To quantify parasite growth while controlling for variability between trials, we calculated the post-invasion parasitemia relative to the parasitemia in a positive control consisting of red blood cells of all ages–we will refer to this measure as the “relative parasitemia”. To estimate parasite selectivity for red blood cell targets, we counted the number of parasites in invaded cells to generate a distribution of multiply infected cells, consistent with previous studies [24]. Importantly, we used a standard static assay to improve our ability to quantify multiply infected cells, a key parameter in the measurement of invasion pathway.

**Fig 1:**
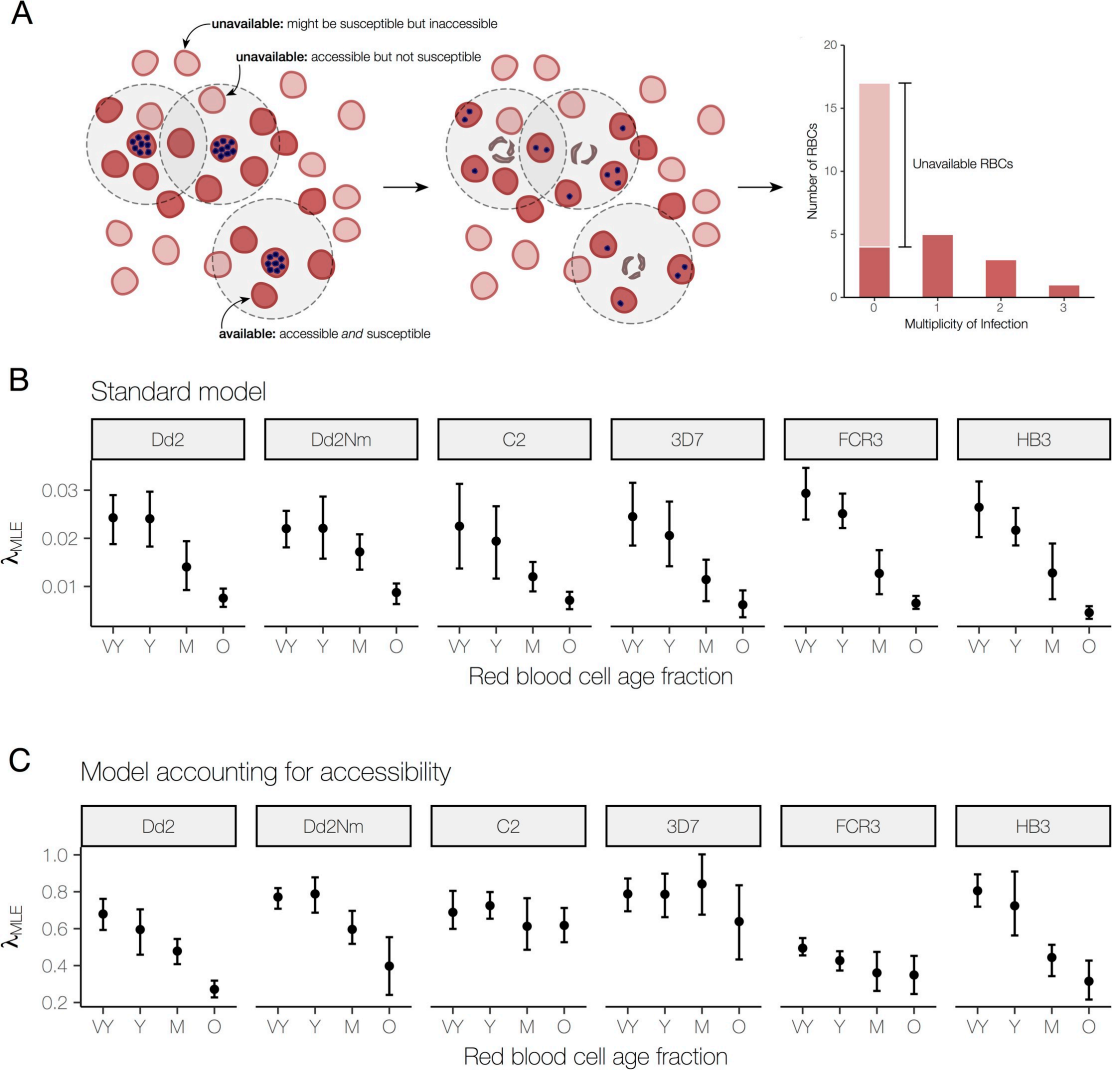
Accounting for availability reveals different responses to red blood cell age. **(A)** Pre-invasion *(left)*: Red blood cells available for invasion (darker red) are both accessible–within the burst radius of a schizont (blue dots represent merozoites)–and susceptible, i.e. possessing the required receptors. Unavailable red blood cells (light red) may be too far from a schizont or within the burst radius but not susceptible. Post-invasion *(middle)*: After schizonts burst, available red blood cells may escape infection or be newly infected with 1 or more merozoites. Distribution of infection multiplicity *(right)*: Post-invasion, we count the number of red blood cells infected with 0, 1, 2, and 3 merozoites to generate a distribution of infection multiplicity. A Poisson rate can be estimated using the distribution that includes all red blood cells or a distribution that considers only available red blood cells (darker red bars). **(B)** Maximum-likelihood estimates of the unadjusted invasion rate (***λ***_***MLE***_) using the standard model. The distributions (mean with 95% bootstrapped CI) of invasion rate estimates (N = 5 to 8 trials), organized by the parasite strain (panel title) and red blood cell age fraction (x-axis, VY = very young, Y = young, M = medium, O = old) used in the invasion assay. **(C)** Maximum-likelihood estimates of the adjusted invasion rate (***λ***_***MLE***_) using the model accounting for accessibility. The distributions are presented in the same way as in (B).

The physical inaccessibility of red blood cells in static cultures relative to continuously agitated cultures can significantly alter measures of parasite growth and selectivity [24]. Using the Boolean-Poisson model from percolation theory, we estimated a vast majority of red blood cells are likely to be inaccessible to invasion in static culture (see S1 Text for estimates of accessibility). To account for the fact that not all cells are both susceptible and physically accessible to invasion, which we will refer to as available for invasion, we modeled the distribution of invasion multiplicity using a zero-inflated Poisson model, which allowed us to jointly estimate how many cells pre-invasion were actually available and the invasion rate into those cells (Fig 1A, Methods). The zero-inflated Poisson model is parameterized by *f* and *λ*, where *f* is the probability that the data are generated from a Poisson process with rate *λ*, and 1−*f* is the probability of a structural zero. In our biological context, *f* is the fraction of red blood cells available for invasion, and *λ* is the invasion rate. Note that the original SI [24] assumed the Poisson as a null model, with all cells being available for invasion. We will refer to invasion rate estimates based on the zero-inflated Poisson model as “adjusted” estimates and those based on a Poisson model as “unadjusted”. We compared the two models using a likelihood ratio test, and for 169 (89%) of the 190 invasion assays performed, the zero-inflated Poisson model gave a significantly better fit than the Poisson model, assuming an overall significance level of 0.05 with Bonferroni correction (S1 Fig). A visual comparison of the predictions given by the two models can be found in the Supporting Information, organized by strain (S5 Fig, S6 Fig, S7 Fig, S8 Fig, S9 Fig and S10 Fig).

Once we accounted for restricted physical access to host targets, we measured increased invasion rates and substantial differences between strains in their ability to invade red blood cells of different ages. The unadjusted estimates of the invasion rate declined with increasing red blood cell age for all laboratory strains (Fig 1B), whereas the adjusted estimates showed that two laboratory strains, C2 and 3D7, maintained a constant invasion rate with increasing red blood cell age (Fig 1C). This suggests that the decreasing trends for these strains using the standard approach were caused by the reduced number of cells being available for invasion in the older fractions rather than reduced efficiency of invasion into them.

The fraction available, *f*, has a natural interpretation as a measure of selectivity when physical accessibility is held constant, e.g. in the same culture conditions: a lower fraction available in the same culture conditions is indicative of a more selective invasion process. Since cells shrink and become more rigid with age, it is likely that there are differences in red blood cell packing–even with the same culture conditions–between age fractions. However, given the finite number of merozoites per schizont and the small change in the overall diameter of cells as they age, we propose that the accessibility of susceptible cells remains similar between age fractions, with invasion efficiency likely to be the main driver of invasion. Our fraction available estimates are therefore related to the SI–which is essentially a measure of how poorly the Poisson model fits the observed data–and we found a strong negative correlation between the fraction susceptible and the SI (Spearman’s rank correlation = -0.95, N = 190). Unlike the SI, however, the fraction available is biologically interpretable and amenable to maximum likelihood statistics (providing confidence intervals) and is thus a rigorous alternative to quantifying selectivity in parasite growth.

### *P*. *falciparum* strains with similar multiplication rates exhibit distinct invasion strategies with respect to red blood cell age

**Fig 2:**
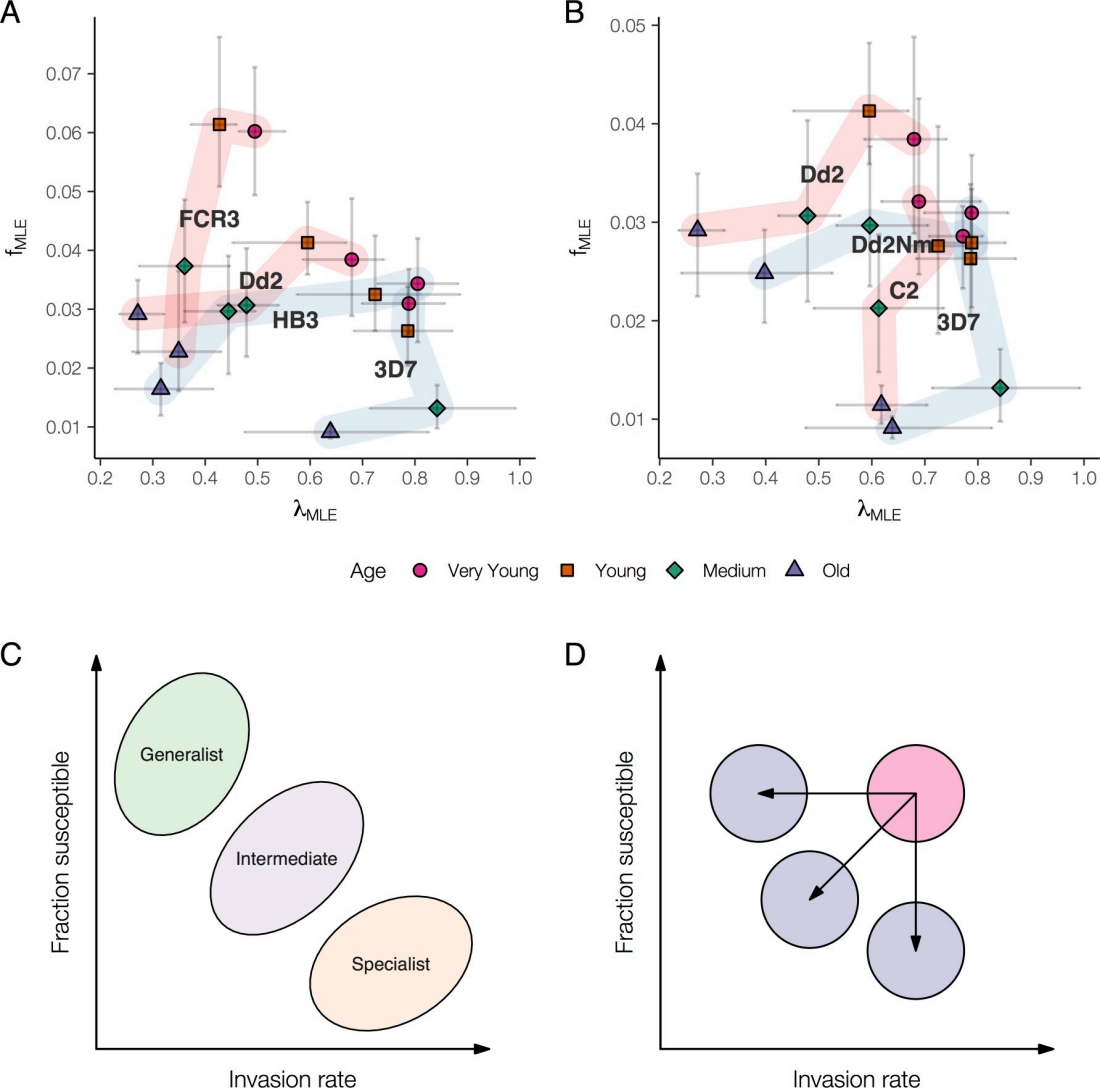
**(A, B)** Modeling availability reveals distinct invasion strategies. Each point denotes the maximum likelihood estimate of the fraction available ***f***_***MLE***_ (y-axis) and ***λ***_***MLE***_ (x-axis), colored (and symbolized) by the age fraction and labelled by the parasite strain used in the invasion assay. Horizontal and vertical bars show the 90% bootstrap confidence intervals. Points for the same strain are connected by a light band in order of increasing red blood cell age. A light red band indicates that the strain is sialic-acid dependent (FCR3, Dd2, C2); blue, sialic-acid independent (HB3, 3D7, Dd2Nm). **(B)** shows four laboratory strains: FCR3, Dd2, HB3, and 3D7. **(C)** shows two isogenic strain pairs, Dd2/Dd2Nm and C2/3D7, where the strains in each pair differ by a single point mutation that is associated with their dependence on sialic-acid receptors. **(C, D)** Conceptualizing invasion strategies and response to red blood cell aging. **(C)** Parasites with similar PMR can be distinguished in terms of how they tradeoff between invasion rate and fraction available, with generalists—low invasion rate into a high fraction available—and specialists—high invasion rate into a low fraction available—on either end of the spectrum. **(D)** Strains can be further distinguished by their response to aging red blood cells—whether they experience a decline in invasion rate (horizontal arrow), a decline in fraction susceptible (vertical arrow), or a combination of both (diagonal arrow).

We mapped *λ* (invasion efficiency) and *f* (fraction available) for four lab strains–Dd2, 3D7, FCR3, and HB3 –with known differences in invasion phenotype, but similar PMR in whole blood in our assays (S2 Fig). Although the relative parasitemia for a given red blood cell fraction was similar between strains (S2 Fig), this was achieved with different tradeoffs between fraction susceptible and invasion rate (Fig 2A, and S3 Fig stratified by age fraction). We propose that these differences may reflect different ecological strategies for invading heterogeneous red blood cells, which we place along the spectrum from generalist to specialist (Fig 2C). The strains exhibit patterns of invasion that are qualitatively consistent with this framework. For example, FCR3 has a generalist invasion strategy: a higher fraction of red blood cells is available for invasion, but the invasion rate into these cells is lower. On the other hand, 3D7 appears to have a more specialist strategy: a lower fraction of red blood cells is available, but the invasion rate into these is higher. Dd2 and HB3 exhibit an intermediate strategy. We also compared two pairs of isogenic laboratory strains, Dd2/Dd2Nm and C2/3D7, where the strains in each pair differ by a single point mutation that renders them SA-dependent/independent, respectively. Fig 2B illustrates that within each pair, the SA-independent strain displayed a more specialist phenotype (i.e. for each red blood cell age fraction, the SA-independent strain had a lower fraction available, but a higher invasion rate than the corresponding SA-dependent strain).

A further distinction can be made with respect to red blood cell aging. Although relative parasitemia declines in older red blood cells for every strain (S4 Fig), the decline occurs in different ways: FCR3 and 3D7 experience a steep drop in fraction available with relatively little change in invasion rate, whereas Dd2 and HB3 experience a smaller decrease in fraction available, but a larger decrease in invasion rate (Fig 2A). The paired isogenic strains had similar responses to red blood cell aging: Dd2 and Dd2Nm had a modest decrease in fraction available, but a larger decrease in invasion rate, whereas C2 and 3D7 had a substantial decrease in fraction available, but a relatively small decrease in invasion rate (Fig 2B). Fig 2D illustrates a conceptual model of these differential responses to cell aging. Of note, *ex vivo* assays of field isolates from clinical malaria cases in Senegal showed variation across these gradients, both in terms of overall strategy (S4A Fig) and in their response to red blood cell aging (S4B Fig).

### Diverse invasion strategies generate important differences in within-host dynamics

**Fig 3:**
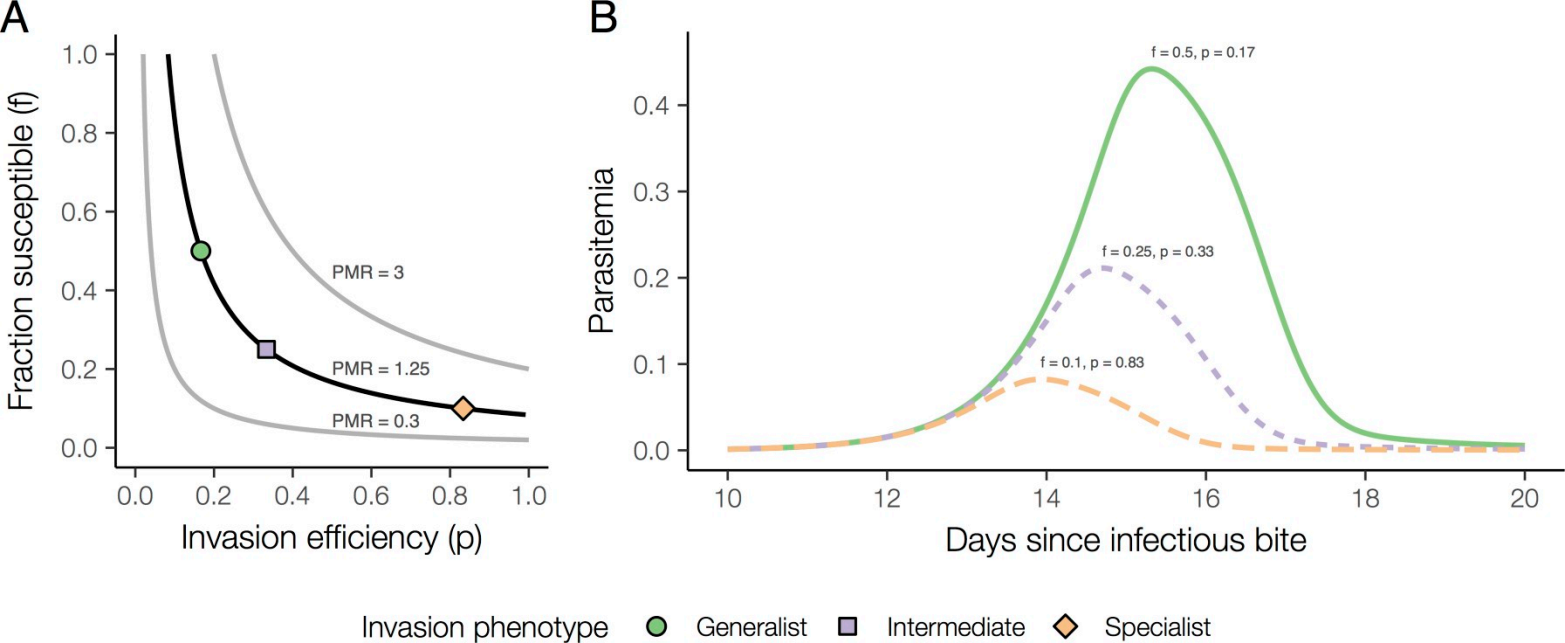
Simulated in vivo invasion dynamics of different invasion strategies. **(A)** The three curves show the inversely proportional relationship between fraction available and invasion efficiency for different fixed values of PMR: 0.3, 1.25, or 3. For our simulations, we considered three invasion phenotypes corresponding to a PMR of 1.25 (black curve): a generalist phenotype (green circle: ***f*** = 0.5, ***p*** = 0.17); an intermediate phenotype (purple square: ***f*** = 0.25, ***p*** = 0.33); and a specialist phenotype (orange diamond: ***f*** = 0.1, ***p*** = 0.83). **(B)** Simulated parasitemia during infection with the specialist (orange long dash), intermediate (purple short dash), or generalist (green line) strain for the period 10–20 days after the infectious bite.

To predict the effects of different invasion strategies on within-host dynamics, we used a simple compartmental model with three red blood cell compartments (susceptible, non-susceptible, and infected) and one merozoite compartment (see Methods). We compared hypothetical strains that achieve the same PMR through different combinations of fraction available (denoted *f*) and invasion efficiency (denoted *p* and defined as the probability that a parasite successfully invaded after coming into contact with a permissive red blood cell). For a fixed PMR, the fraction available and invasion efficiency are inversely proportional (Fig 3A). We included a specialist strain (*f* = 0.1, *p* = 0.83), a generalist strain (*f* = 0.5, *p* = 0.17), and an intermediate strain (*f* = 0.25, *p* = 0.33). Despite all three strains having the same PMR, the generalist strain had the highest peak parasitemia, and the specialist strain had the lowest (Fig 3B). Thus, we predict that PMR may not provide information about the proliferation potential of a parasite strain and that–consistent with some, but not all previous observations of SI–specialization for particular red blood cell age subpopulations may lead to lower peak parasite densities *in vivo*.

## Discussion

Using a new approach to understand the nuances of parasite invasion *in vitro*, we have shown that laboratory lines that would have otherwise been indistinguishable using standard approaches exhibit distinct invasion strategies with respect to the same red blood cell age fraction. Based on our findings, we propose that in addition to pressures from the immune system (41,42), variations in invasion pathway utilization across *P*. *falciparum* strains may represent an adaptation to the heterogeneous and dynamic ecological landscape of the host blood stream.

Availability for invasion requires that red blood cells be physically accessible to merozoites, which are non-motile, as well as susceptible (i.e. expressing receptors required by the parasite). Fitting a Poisson distribution assumes that all red blood cells are equally available and that invasion occurs randomly. In reality, however, a number of factors, such as immunity, rosetting, and differential susceptibility of red blood cells, cause deviations from this model. The SI has been used by some researchers to measure heterogeneous invasion *in vitro* [24,37,38], but by assuming that all red blood cells are accessible, is unable to detect more subtle trends in susceptibility. To address this issue, we used a zero-inflated Poisson model that explicitly models availability. This is particularly useful to *in vitro* static cultures, where experimental conditions render a significant fraction of cells physically inaccessible to the parasite. When experimental conditions, and thus physical accessibility, are held constant, the estimated fraction available is indicative of the relative susceptibility of different subpopulations of red blood cells to different parasite strains.

By disentangling these different aspects of invasion, availability and invasion rate, we were able to distinguish between generalist and specialist *P*. *falciparum* strains. The former is able to invade a larger fraction of red blood cells, but at a lower rate, whereas the latter is able to invade a smaller fraction of red blood cells, but at a higher rate. We have found that even with a pooling of all red blood cell age fractions, the difference between a generalist and a specialist strain remains. Using a compartmental model, we have shown that this has consequences *in vivo*: in comparison with a specialist strain with the same PMR, a generalist strain is likely to exhibit higher parasite densities, which may in turn affect disease severity. This is supported by the work of Lim et al., which suggested that adaption of *P*. *knowlesi* to a broader age range of red blood cells is the likely the cause of higher parasitemia *in vivo*; this adaptation was found to persist for many generations in parasites cultured *in vitro* [39]. One important feature of natural malaria infections that could lead to different invasion strategies is the presence of multiple genotypes infecting the same host.

Conversely, modeling work by Kerlin et al. has shown that a restricted age range of susceptible red blood cells is a credible mechanism by which parasites may self-regulate proliferation [40]. The importance of red blood cell age in predicting infection dynamics within a host has been modeled for human malaria [41] as well as rodent malaria [42,43]. Of note, the impact of the heterogeneous susceptibility of cells for infection dynamics is paralleled in models of an infectious disease spreading through an age-structured population of hosts, where epidemic trajectory depends not on the R_0_ overall, but on the R_0_ in different subgroups of the population [44].

For R_0_ calculations, as with our framework, transmission is determined by both the contacts between infectious and susceptible individuals (analogous in some models to our measure of accessibility) and the probability of invasion given contact (analogous to invasion efficiency). There are intrinsic difficulties in disentangling the relationship between these two components of transmission, and theoretical epidemiological models have shown that the structure of the contact network, in addition to the heterogeneity in the kinds of contact, is important: i.e. how many contacts does an infectious person have? We have in effect assumed that by using static cultures but changing the age of the cells, we are maintaining similar contacts structures, such that we can observe differences in invasion efficiency. There is a formal possibility that cells of different age pack differently in static cultures, however, altering the contact structure itself, and confounding invasion efficiency differences. More work is needed to measure the structural aspects of red blood cell culture if we are to control for differences in cell packing using approaches like these, and single cell approaches may be able to provide even more specific insights into the distribution of heterogeneities in cell susceptibility to parasite invasion.

Our findings not only have implications for how we measure and understand the relationship between invasion phenotypes and virulence, but also how we predict the impact of the skewing of red blood cell age structure due to anemia and measure growth parameters *in vitro*. Our adjusted methods for measuring these aspects of parasite invasion therefore provide a new tool to assess the strategies used among field isolates in different clinical and transmission settings.

## Materials and methods

### Density-dependent fractionation of red blood cells

Red blood cells of different ages were fractionated by exploiting density differences among red blood cell subpopulations using a discontinuous Percoll gradient as previously described [45–48]. In brief, blood was incubated at 37°C for 30 hours prior to fractionation [49]. Five Percoll solutions of different concentrations (40%, 45%, 50%, 53.5%, and 65%) were diluted in un-supplemented RPMI. Two mL of each dilution were overlaid into a 15 mL Falcon tube from most dense (65%) to least dense (40%). 500 μL of a 30% hematocrit suspension of washed O^+^ blood cells was then added. The gradient was centrifuged for 12 min at 1200 x g, at 22°C. Four prominent bands corresponding to red blood cells of different ages (very young, young, medium and old) were resolved, with the densest (oldest) cells settling into the bottommost layer. Although the relative abundance of each age fraction varied by blood donor, the approximate proportions of red blood cell fractions were as follows: 40%, 30%, 20%, and 10% for very young, young, medium, and old, respectively.

### ADVIA analysis

An ADVIA hematological analyzer (Bayer) was used to validate the efficacy of fractionation of whole blood into discrete age groups. Following Percoll fractionation, each of the red blood cell subpopulations was washed and re-suspended in 1x PBS. Measurements were taken for each age fraction, as well as a positive control consisting of Percoll-fractionated blood that was subsequently re-pooled. Older cells were associated with decreased cell size (mean corpuscular volume [MCV]) and increased density (mean corpuscular hemoglobin concentration [MCHC]), demonstrating the isolation of distinct red blood cell subpopulations [50].

### Age-dependent invasion assay

Age-dependent invasion efficiencies were measured using standard invasion assays. Invasion assays were performed on sorbitol-synchronized, ring-stage parasites plated at a final parasitemia of 0.7% and a hematocrit of 2%. Parasitized donor cells were added to an equal volume of age-fractionated (very young, young, medium, and old) red blood cells (receptor cells) in a 96-well plate. Donor cells were treated with both neuraminidase (Calbiochem, 66 mU/ml) and chymotrypsin (Worthington Biochemicals, 1 mg/ml) for 1 hour at 37°C. This enzyme treatment cleaves the majority of known red blood cell receptors and renders these cells refractory to parasite invasion, ensuring that only age-fractionated receptor cells are susceptible to invasion. The negative control was composed of receptor and donor cells that were enzyme-treated with neuraminidase and chymotrypsin. The positive control consisted of Percoll-fractionated cells that were subsequently re-pooled (and therefore representative of whole blood), but subjected to the same manipulations as each of the individual red blood cell fractions. Samples were plated in triplicate and incubated at 37°C for 48 hours until parasite re-invasion. Blood smears were then made and parasitemia determined microscopically. Assays were repeated a minimum of five times.

### Statistical analysis of invasion data

Parasitemia was calculated for each assay and divided by the values in the positive control to account for variation between trials to yield the “relative parasitemia”. Since the pre-invasion parasitemia was fixed, the post-invasion parasitemia was proportional to the PMR, and the relative parasitemia was equivalent to the relative PMR (using the positive control as the reference). With relative parasitemia as the outcome variable, we examined the effect of red blood cell age on each strain and the effect of parasite strain within each age group using the non-parametric Kruskal-Wallis test. To account for multiple tests across strata of different red blood cell age groups/parasite strains, the Bonferroni correction was applied. Significant Kruskal-Wallis test results were followed by a post-hoc Dunn’s test to examine pairwise differences between groups. A Type I error rate of 0.05 was used to determine statistical significance. The analysis was performed using R 3.3.3 and the R package dunn.test [51,52]. All six strains exhibited heterogeneity across the different age fractions (Kruskal-Wallis rank sum test, p < 0.01 for all strains), with lower relative parasitemia in older age fractions (S4 Fig). However, within each of the four age fractions, no significant heterogeneity was detected between strains (Kruskal-Wallis rank sum test, p = 0.35, very young; p = 0.94, young; p = 0.43, medium; 0.07, old, S2 Fig).

### Zero-inflated Poisson model of infection multiplicity

In each invasion assay, the number of parasites in each red blood cell (i.e. its multiplicity of infection [MOI]) was determined by microscopy for roughly 300 infected cells. For each MOI, we counted the number of red blood cells with that MOI. The maximum MOI observed was 5. The number of uninfected red blood cells (i.e. having an MOI of 0), was estimated from parasitemia. These counts were used to fit a zero-inflated Poisson model parameterized by *f* and *λ*, where *f* is the probability that the data were generated from a Poisson process with rate *λ*, and 1−*f* is the probability of a structural zero. Maximum likelihood estimates of the parameters were calculated. A Poisson model with no zero inflation (i.e. *f* = 1) was also fit to the data using maximum likelihood estimation, and the likelihood ratio test was used to compare the zero-inflated and non-zero-inflated models.

### Within-host infection dynamics compartmental model

A continuous-time compartmental model was used to simulate within-host infection dynamics. Four compartments were used: three to track the numbers of susceptible, unsusceptible, and infected red blood cells, and one to track the number of merozoites. We assumed that all cells are accessible *in vivo*–thus, the fraction available is equal to the fraction susceptible. *The* rate of production of new red blood cells was chosen to maintain 20–30 trillion red blood cells in circulation absent infection [53], given a red blood cell lifespan of 110 days [54]. Of the new red blood cells, a constant fraction entered as susceptible red blood cells. We assumed that at the start of infection, inoculation with sporozoites resulted in 15 liver-stage parasites, each of which released 40,000 merozoites into circulation [55–58]. For simplicity, the rate of infection assumed merozoites contacted a susceptible red blood cell every 10 minutes, resulting in successful invasion with a certain probability that varied between simulations. An infected red blood cell has a lifespan of 2 days, corresponding to the duration of schizogony [59,60]. The fraction of red blood cells susceptible and the probability of infection given contact were varied between simulations. This model is described in more detail in the Supporting Information (S1 Text).

### Ethics statement

This study was approved by the Institutional Review Board of the Harvard School of Public Health (CR-16330-01) and by the Ethics Committee of the Ministry of Health in Senegal (0127MSAS/DPRS/CNRES).

## Supporting information

S1 Fig Comparison of Poisson and zero-inflated Poisson model fits. For each invasion assay, the empirical distribution of the number of parasites in a red blood cell was used to fit a Poisson model and a zero-inflated Poisson model. Model fits were compared using a likelihood ratio test. The p-values (N = 190, y-axis, negative log-transformed) are plotted above, organized by the parasite strain (panel title) and red blood cell age fraction (x-axis) used in the invasion assay. The abbreviations used in the x-axis are: P (pooled), VY (very young), Y (young), M (medium), O (old). For each combination of strain and age, multiple p-values, from different trials, are shown with their horizontal position jittered. The horizontal dashed lines in each panel are the significance cutoff assuming an overall significance level of 0.05 and Bonferroni correction. P-values lying below the line (highlighted in red) correspond to trials for which the zero-inflated Poisson model did not provide not a significantly better fit than the Poisson model.
(PDF)

S2 Fig Relative post-invasion parasitemia versus strains. The distribution (mean with 95% bootstrap CI) of post-invasion parasitemia in each age fraction relative to pooled blood. The distributions are organized by strain (x-axis) and age fraction (panel title). For all age fractions, the Kruskal-Wallis rank sum test did not detect significant heterogeneity between strains (from youngest to oldest, p = 0.42, 0.94, 0.47, and 0.09).
(PDF)

S3 Fig Maximum likelihood estimate of the fraction susceptible *f_MLE_* (y-axis) and *λ_MLE_* (x-axis) for six lab strains, stratified by age of red blood cell fraction. (PDF)

S4 Fig Invasion strategies of field strains. **(A)** Points show the maximum likelihood estimate of the fraction susceptible ***f***_***MLE***_ (y-axis) and ***λ***_***MLE***_ (x-axis) for field strains cultured *ex vivo* in each of four red blood cell age fractions (panel titles). For some trials, the model could not be fit due to small numbers of infected cells; there are 20, 18, 16, and 12 strains shown in the very young, young, medium, and old panels. **(B) *f***_***MLE***_ (y-axis) and ***λ***_***MLE***_ (x-axis) from (A) for the very young (pink) and old (blue) red blood cell age fractions are paired by strain here to highlight strain-specific responses to red blood cell aging.
(PDF)

S5 Fig Distribution of multiplicity used to estimate invasion profile of the 3D7 strain. The frequency of multiply infected cells post-invasion is shown for pooled (P), very young (VY), young (Y), medium (M), and old (O) red blood cells, for each replicate (22–27). We compare the observed number (green) to the Poisson prediction (orange) and the zero-inflated Poisson prediction (purple).
(PDF)

S6 Fig Distribution of multiplicity used to estimate invasion profile of the C2 strain. The frequency of multiply infected cells post-invasion is shown for pooled (P), very young (VY), young (Y), medium (M), and old (O) red blood cells, for each replicate (22–27). We compare the observed number (green) to the Poisson prediction (orange) and the zero-inflated Poisson prediction (purple).
(PDF)

S7 Fig Distribution of multiplicity used to estimate invasion profile of the Dd2 strain. The frequency of multiply infected cells post-invasion is shown for pooled (P), very young (VY), young (Y), medium (M), and old (O) red blood cells, for each replicate (22–27). We compare the observed number (green) to the Poisson prediction (orange) and the zero-inflated Poisson prediction (purple).
(PDF)

S8 Fig Distribution of multiplicity used to estimate invasion profile of the Dd2Nm strain. The frequency of multiply infected cells post-invasion is shown for pooled (P), very young (VY), young (Y), medium (M), and old (O) red blood cells, for each replicate (22–27). We compare the observed number (green) to the Poisson prediction (orange) and the zero-inflated Poisson prediction (purple).
(PDF)

S9 Fig Distribution of multiplicity used to estimate invasion profile of the FCR3 strain. The frequency of multiply infected cells post-invasion is shown for pooled (P), very young (VY), young (Y), medium (M), and old (O) red blood cells, for each replicate (22–27). We compare the observed number (green) to the Poisson prediction (orange) and the zero-inflated Poisson prediction (purple).
(PDF)

S10 Fig Distribution of multiplicity used to estimate invasion profile of the HB3 strain. The frequency of multiply infected cells post-invasion is shown for pooled (P), very young (VY), young (Y), medium (M), and old (O) red blood cells, for each replicate (22–27). We compare the observed number (green) to the Poisson prediction (orange) and the zero-inflated Poisson prediction (purple).
(PDF)

S1 Text Additional modeling details. Mathematical descriptions of the compartmental model used to simulate within-host infection dynamics and the Boolean-Poisson model used to estimate physical accessibility.
(DOCX)
