# Supplementary figures and images for: Accounting for red blood cell accessibility reveals distinct invasion strategies in Plasmodium falciparum strains
Source: PLoS Comput Biol. 2020 Apr 21;16(4):e1007702. doi: 10.1371/journal.pcbi.1007702 (PMC7194430; doi:10.1371/journal.pcbi.1007702)

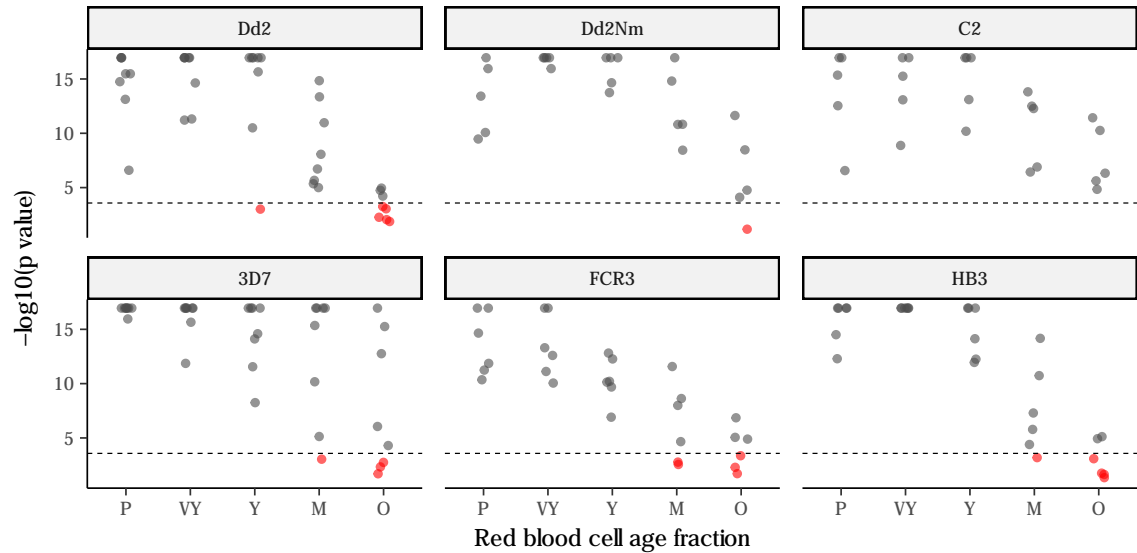

Supplement: S1 Fig — For each invasion assay, the empirical distribution of the number of parasites in a red blood cell was used to fit a Poisson model and a zero-inflated Poisson model. Model fits were compared using a likelihood ratio test. The p-values (N = 190, y-axis, negative log-transformed) are plotted above, organized by the parasite strain (panel title) and red blood cell age fraction (x-axis) used in the invasion assay. The abbreviations used in the x-axis are: P (pooled), VY (very young), Y (young), M (medium), O (old). For each combination of strain and age, multiple p-values, from different trials, are shown with their horizontal position jittered. The horizontal dashed lines in each panel are the significance cutoff assuming an overall significance level of 0.05 and Bonferroni correction. P-values lying below the line (highlighted in red) correspond to trials for which the zero-inflated Poisson model did not provide not a significantly better fit than the Poisson model. (PDF) [file pcbi.1007702.s001.pdf]

Relative parasitemia

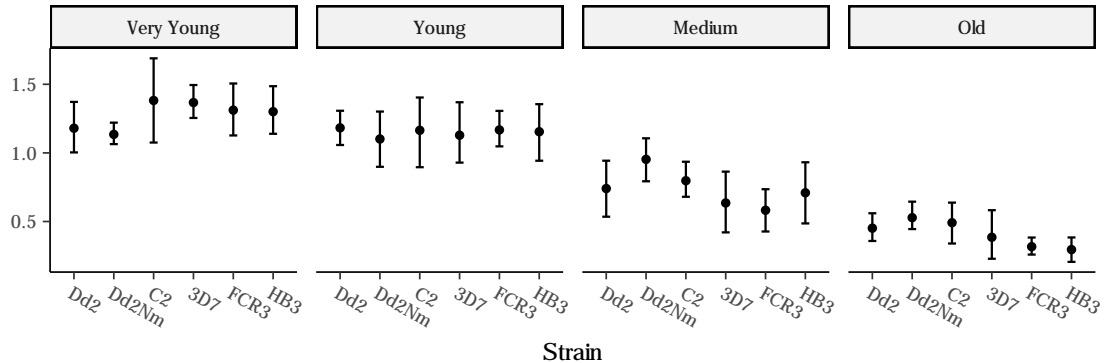

Supplement: S2 Fig — The distribution (mean with 95% bootstrap CI) of post-invasion parasitemia in each age fraction relative to pooled blood. The distributions are organized by strain (x-axis) and age fraction (panel title). For all age fractions, the Kruskal-Wallis rank sum test did not detect significant heterogeneity between strains (from youngest to oldest, p = 0.42, 0.94, 0.47, and 0.09). (PDF) [file pcbi.1007702.s002.pdf]

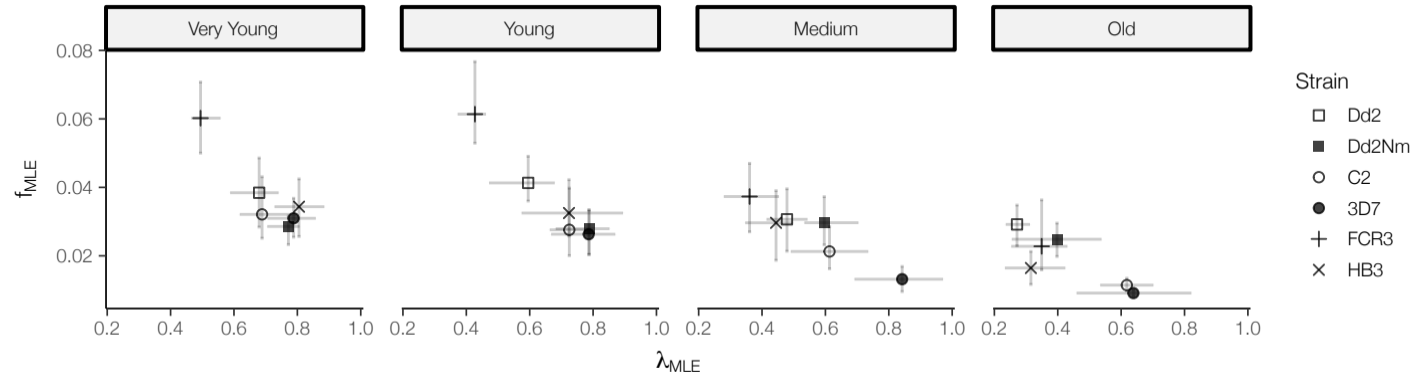

Supplement: S3 Fig — (PDF) [file pcbi.1007702.s003.pdf]

A

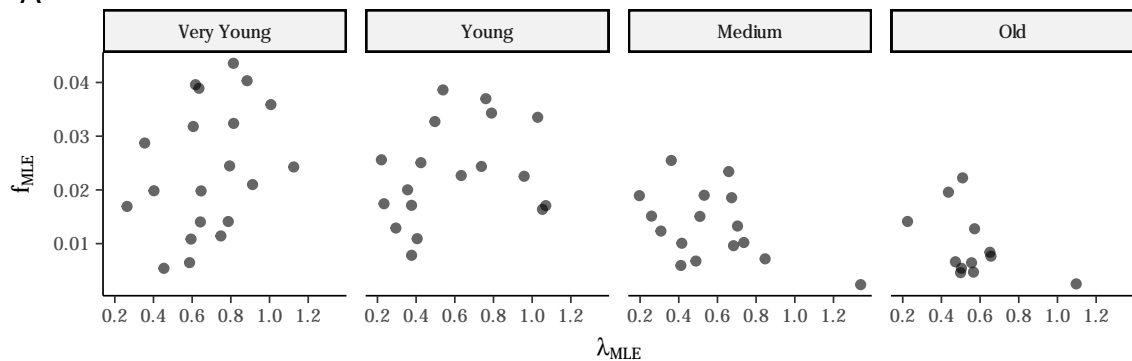

B

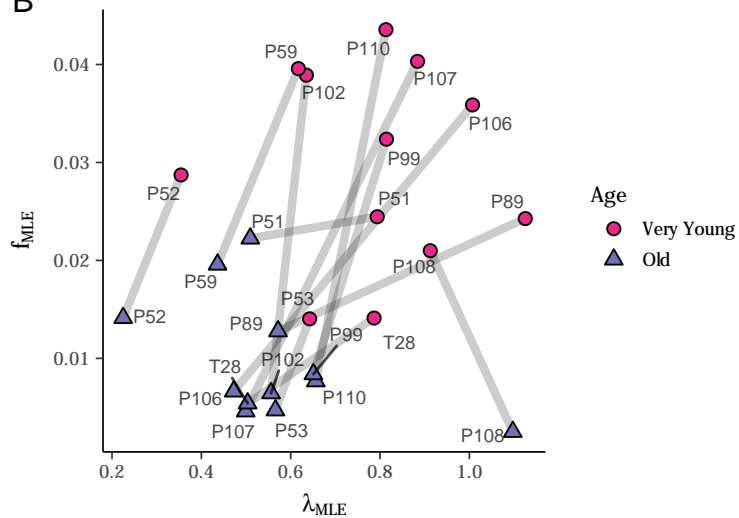

Supplement: S4 Fig — (A) Points show the maximum likelihood estimate of the fraction susceptible fMLE (y-axis) and λMLE (x-axis) for field strains cultured ex vivo in each of four red blood cell age fractions (panel titles). For some trials, the model could not be fit due to small numbers of infected cells; there are 20, 18, 16, and 12 strains shown in the very young, young, medium, and old panels. (B) fMLE (y-axis) and λMLE (x-axis) from (A) for the very young (pink) and old (blue) red blood cell age fractions are paired by strain here to highlight strain-specific responses to red blood cell aging. (PDF) [file pcbi.1007702.s004.pdf]

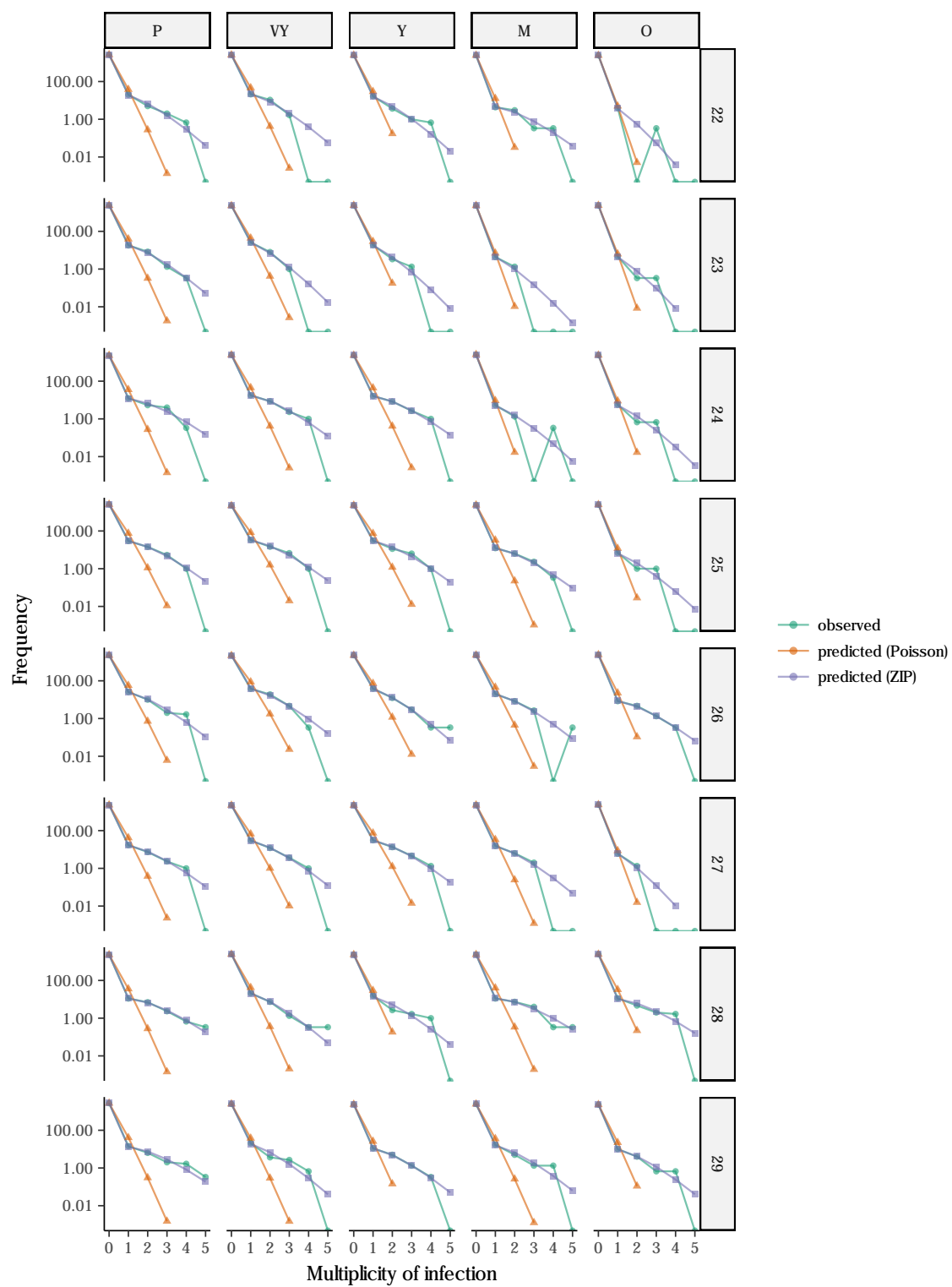

Supplement: S5 Fig — The frequency of multiply infected cells post-invasion is shown for pooled (P), very young (VY), young (Y), medium (M), and old (O) red blood cells, for each replicate (22–27). We compare the observed number (green) to the Poisson prediction (orange) and the zero-inflated Poisson prediction (purple). (PDF) [file pcbi.1007702.s005.pdf]

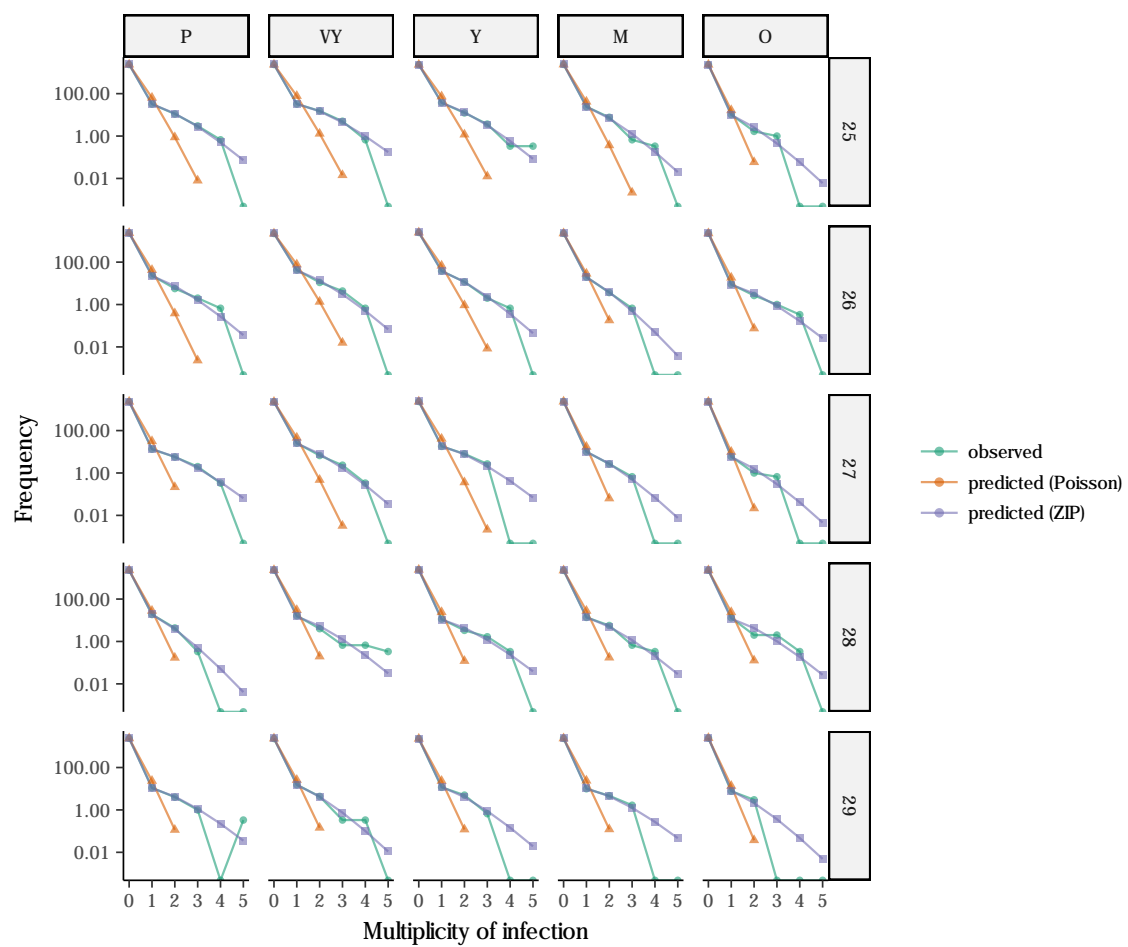

Supplement: S6 Fig — The frequency of multiply infected cells post-invasion is shown for pooled (P), very young (VY), young (Y), medium (M), and old (O) red blood cells, for each replicate (22–27). We compare the observed number (green) to the Poisson prediction (orange) and the zero-inflated Poisson prediction (purple). (PDF) [file pcbi.1007702.s006.pdf]

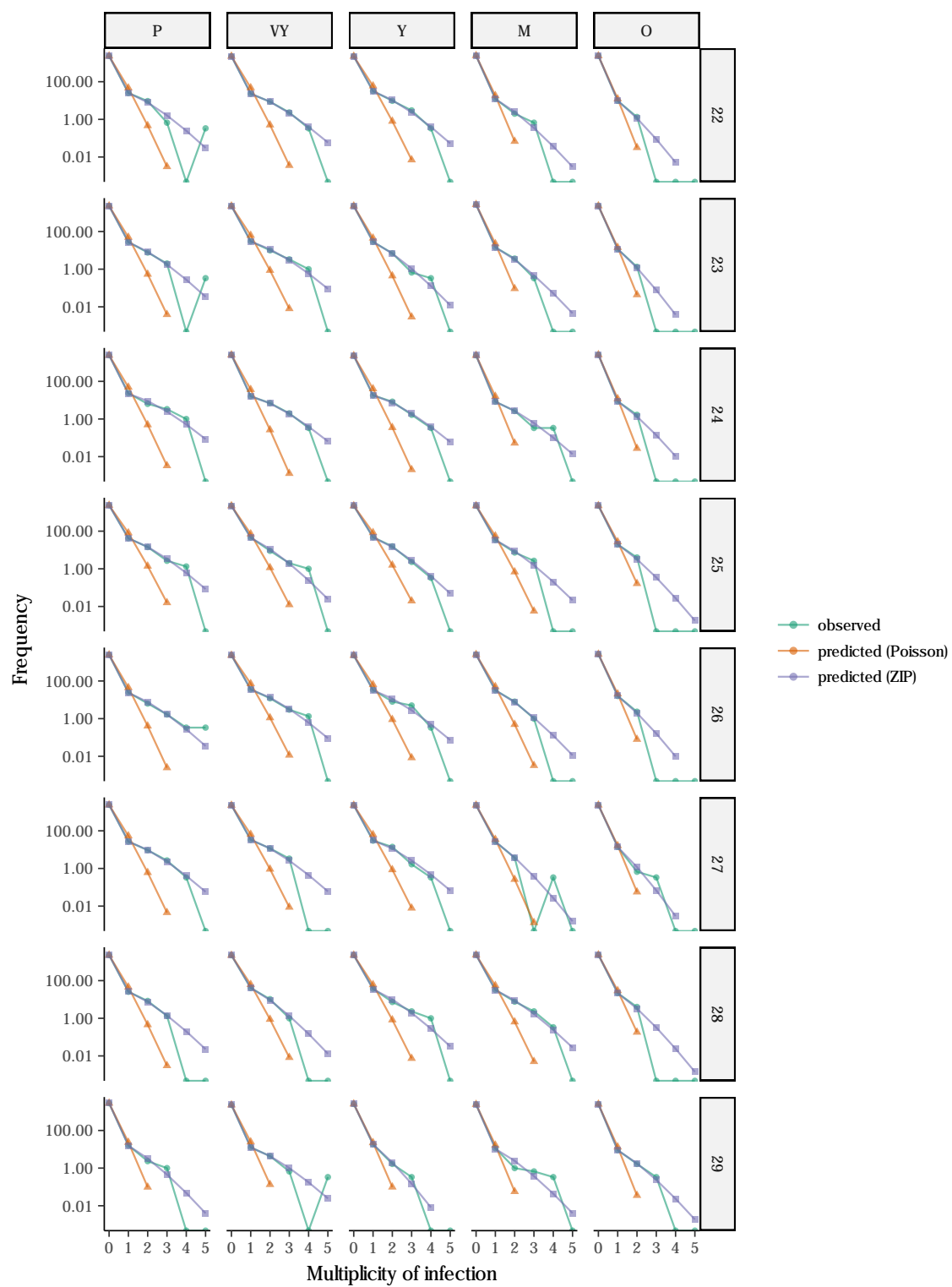

Supplement: S7 Fig — The frequency of multiply infected cells post-invasion is shown for pooled (P), very young (VY), young (Y), medium (M), and old (O) red blood cells, for each replicate (22–27). We compare the observed number (green) to the Poisson prediction (orange) and the zero-inflated Poisson prediction (purple). (PDF) [file pcbi.1007702.s007.pdf]

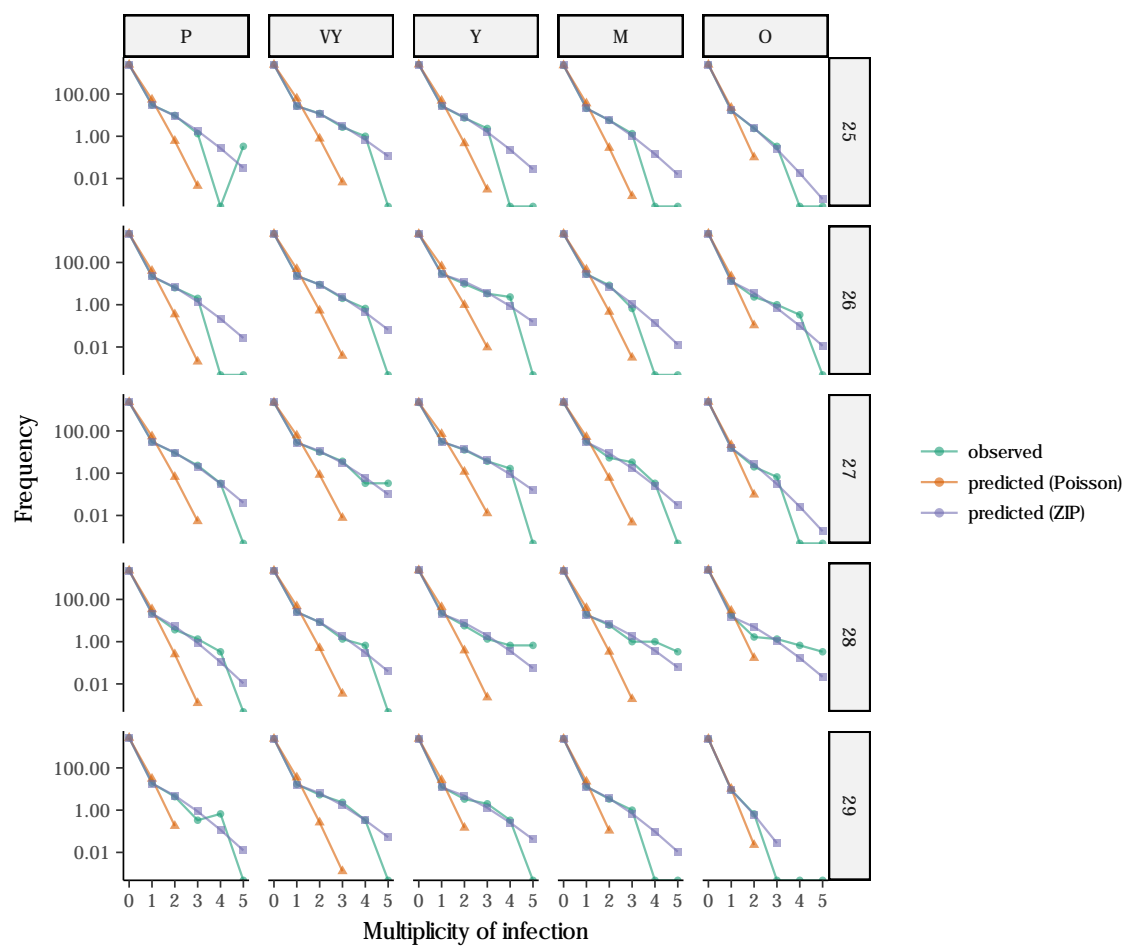

Supplement: S8 Fig — The frequency of multiply infected cells post-invasion is shown for pooled (P), very young (VY), young (Y), medium (M), and old (O) red blood cells, for each replicate (22–27). We compare the observed number (green) to the Poisson prediction (orange) and the zero-inflated Poisson prediction (purple). (PDF) [file pcbi.1007702.s008.pdf]

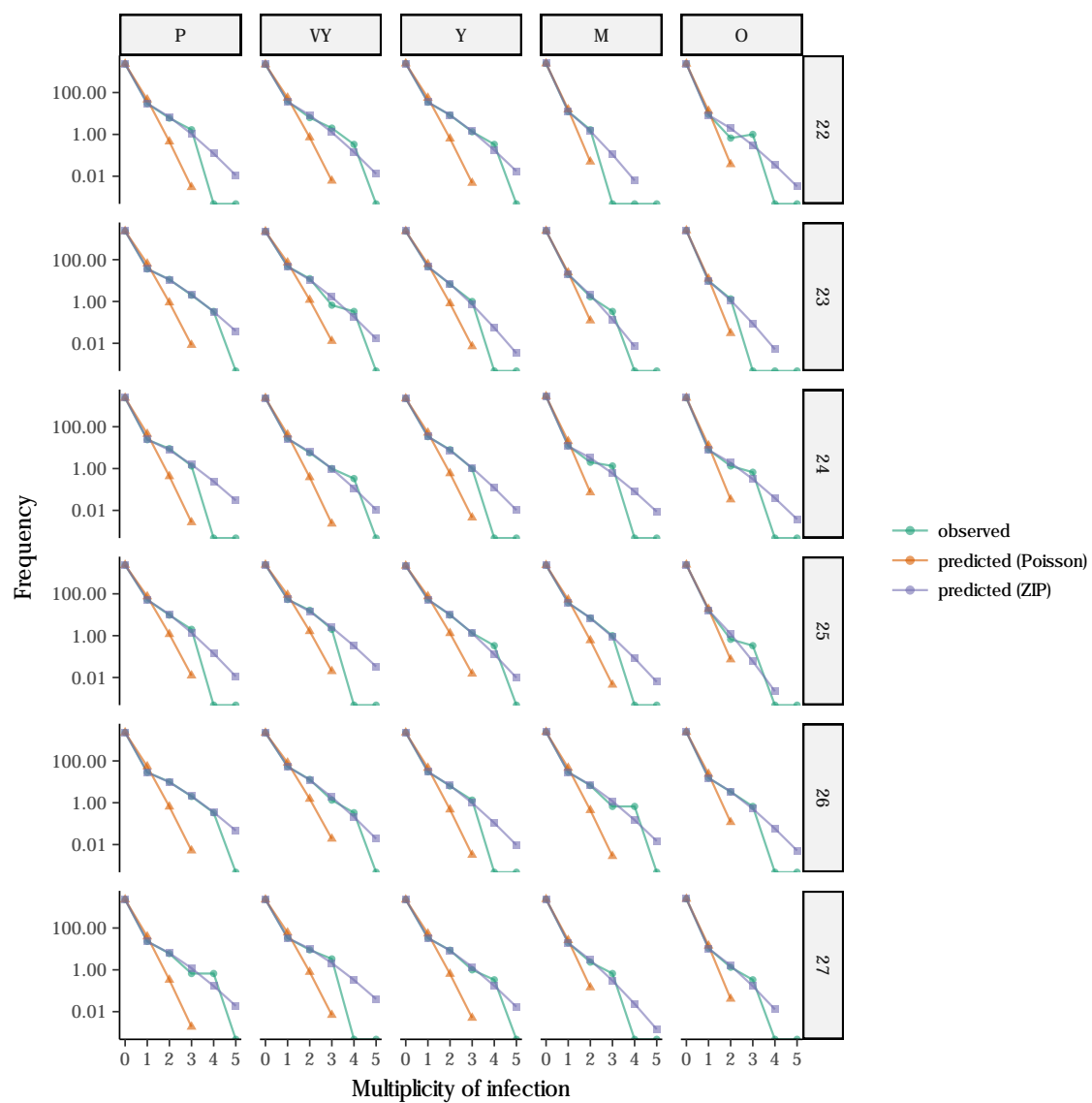

Supplement: S9 Fig — The frequency of multiply infected cells post-invasion is shown for pooled (P), very young (VY), young (Y), medium (M), and old (O) red blood cells, for each replicate (22–27). We compare the observed number (green) to the Poisson prediction (orange) and the zero-inflated Poisson prediction (purple). (PDF) [file pcbi.1007702.s009.pdf]

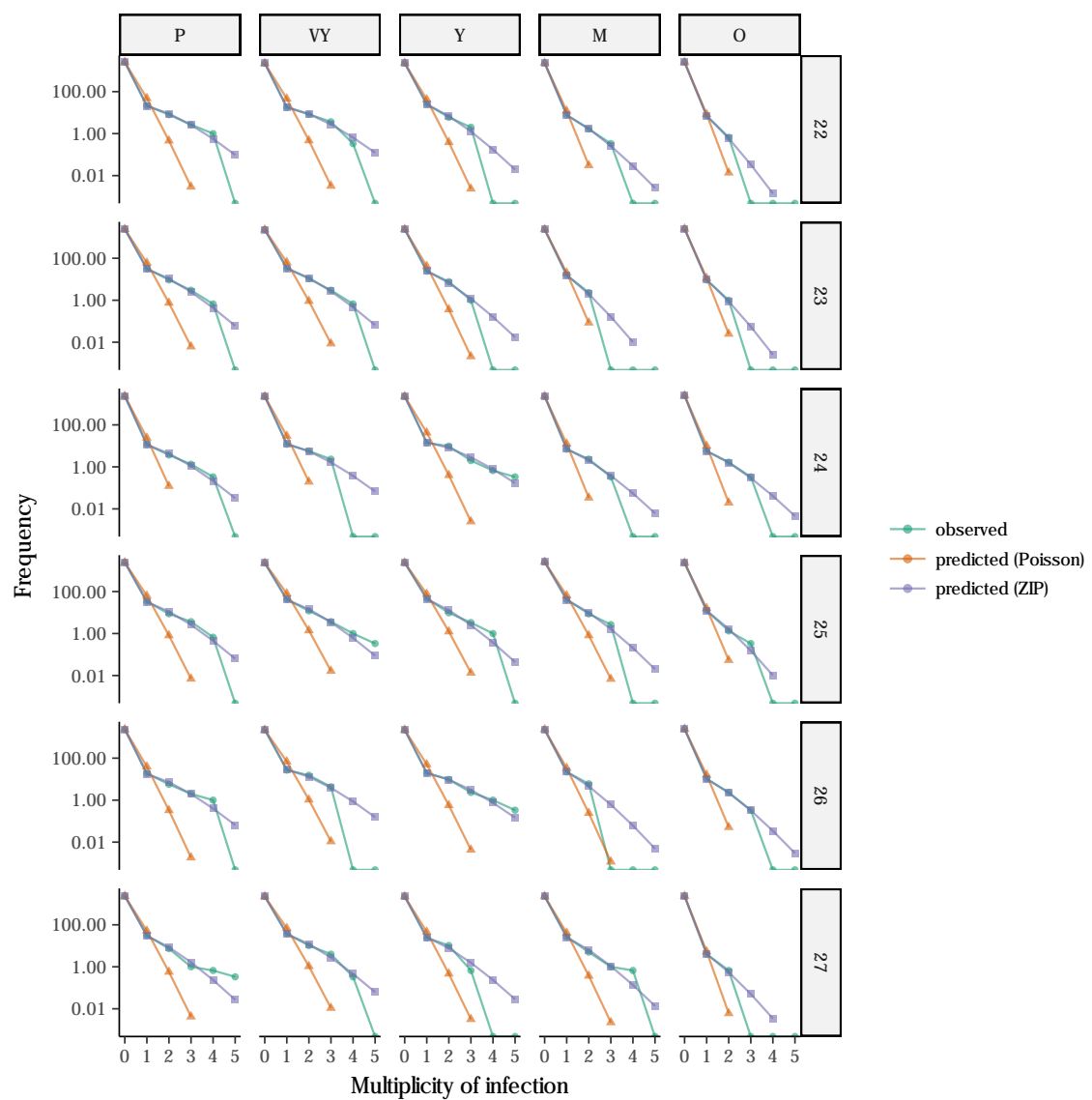

Supplement: S10 Fig — The frequency of multiply infected cells post-invasion is shown for pooled (P), very young (VY), young (Y), medium (M), and old (O) red blood cells, for each replicate (22–27). We compare the observed number (green) to the Poisson prediction (orange) and the zero-inflated Poisson prediction (purple). (PDF) [file pcbi.1007702.s010.pdf]
