# Supplementary material for: Accounting for red blood cell accessibility reveals distinct invasion strategies in Plasmodium falciparum strains
Source: PLoS Comput Biol. 2020 Apr 21;16(4):e1007702. doi: 10.1371/journal.pcbi.1007702 (PMC7194430; doi:10.1371/journal.pcbi.1007702)
Supplement: S1 Text — Mathematical descriptions of the compartmental model used to simulate within-host infection dynamics and the Boolean-Poisson model used to estimate physical accessibility. (DOCX) [file pcbi.1007702.s011.docx]

# Supporting Information

## Within-host Infection Dynamics Compartmental Model Additional Details

A continuous-time compartmental model was used to simulate within-host infection dynamics. The basic model consisted of three red blood cell compartments – susceptible, unsusceptible, and infected (denoted $S$, $R$, and $I$, respectively) – and one merozoite compartment, denoted $Z$. We assumed that *in vivo*, all susceptible cells were accessible. The corresponding system of ordinary differential equations (ODEs) is:

$$S^{'}=f\omega-\beta ZS-\mu S$$

$$R^{'}=\left( 1-f \right)\omega-\mu R$$

$$I^{'}=\beta ZS-\mu_{I}I$$

$$Z^{'}=m\mu_{I}I-\beta ZS- \mu_{Z}Z$$

$\omega$ is the rate of production of new red blood cells, of which fraction $f$ are susceptible to invasion. $\mu$ is the death rate shared by susceptible and unsusceptible red blood cells; $\mu_{I}$ is the death rate for infected red blood cells; $\mu_{Z}$ is for merozoites. $\beta$ is the rate of new infections per susceptible red blood cell per merozoite. $m$ is the number of merozoites released from an infected red blood cell when it ruptures. Parameter values are listed in Supplemental Table 1. For the basic model, the waiting time in each compartment followed an exponential distribution. To more realistically model the lifespan of uninfected and infected red blood cells (approximately 110 days and 2 days, respectively), each red blood cell compartment was replaced with a series of successive compartments, so that the total time spent in a compartment series followed a gamma distribution [61]. $n$ compartments was used to replace the uninfected compartments, and $n_{I}$ compartments was used to replace the infected compartment, resulting in a modified system of ODEs used in our simulations:

$$S_{1}^{'}=f\omega-\beta ZS_{1}-n\mu S_{1}$$

$$S_{k}^{'}=n\mu S_{k-1}-\beta ZS_{j}-n\mu S_{k}, \text{for }k=2, 3, \ldots,n$$

$$R_{1}^{'}=\left( 1-f \right)\omega-n\mu R_{1}$$

$$R_{k}^{'}=n\mu R_{k-1}-n\mu R_{k}, \text{for }k = 2, 3, \ldots, n$$

$$I_{1}^{'}=\beta Z\left( \Sigma_{k}S_{k} \right)-n_{I}\mu_{I}I_{1}$$

$$I_{k}^{'}=n_{I}\mu_{I}I_{k-1}-n_{I}\mu_{I}I_{k}, \text{for }k = 2, 3, \ldots, n_{I}$$

$$Z^{'}= m\mu_{I}\left( \Sigma_{k}I_{k} \right)-\beta Z\left( \Sigma_{k}S_{k} \right)- \mu_{Z}Z$$

To choose parameter values given a desired PMR, we used the relationship PMR $\cong mfp$, where $m$ and $f$ are as described above, and $p$ is the probability of invasion given contact between a merozoite and a susceptible red blood cell. This is a reasonable approximation of PMR when there are relatively few multiply-infected red blood cells. We then calculated $\beta$ as $p/\tau$, where $\tau$ is the length of time needed for the merozoite to complete invasion. Values of $m$ and $\tau$ were determined from the literature. For a fixed PMR, choosing a value for $f$ determines the value of $p$, which in turn determines the corresponding value of $\beta$ to be used in the simulation.

For each set of parameter values, the model was simulated for 500 days without merozoites to reach equilibrium, after which merozoites were introduced to the model. We assumed that infection began with 15 infected hepatocytes releasing 40,000 merozoites each, and we continued the simulation for 25 additional days, long enough to observe the first peak parasitemia [55-58,62,63].

## Estimation of Physical Accessibility using a Boolean-Poisson Model

We estimated the fraction of red blood cells accessible to parasite invasion using the Boolean-Poisson model from continuum percolation theory. To use this model, we assumed that the locations of red blood cells and schizonts were random (i.e. can be modelled with a spatial Poisson processes) and that when a schizont burst, the released merozoites are able to access red blood cells within a sphere of fixed radius (the dispersion radius) centered on the location of the bursting schizont. The expected volume fraction accessible by merozoites is then given by

$E\left[ V \right]= 1-\exp\left( -\left( 4/3 \right)\pi R^{3}N \right)$,

where *R* is the dispersion radius, and *N* is the average number of schizonts per unit volume, which can be calculated as the product of red blood cell count (number of red blood cells per unit volume) and schizontemia (number of schizonts per red blood cell, denoted $s$). The red blood cell count itself can then be decomposed as the hematocrit (volume fraction occupied by red blood cells, denoted $h$) divided by the MCV. For simplicity, we assumed that red blood cells are spherical with radius $r$. We can rewrite the number of schizonts in terms of the hematocrit, MCV, and schizontemia and assuming that red blood cells are roughly spherical:

$$N=\text{ }\left( \text{red blood cell}\text{ count} \right)s=\frac{h}{\text{MCV}}s=\frac{h}{4\pi r^{3}/3}s.$$

Substituting this expression into our previous equation for the expected volume fraction gives

$E\left[ V \right]= 1-\exp\left( -\left( R/r \right)^{3}hs \right)$,

where we have written the expected volume fraction accessible as a function of the dispersion radius (relative to the red blood cell radius), hematocrit, and schizontemia. Finally, we assumed that red blood cells are distributed randomly in space, so the expected fraction of volume accessible is also the fraction of red blood cells accessible. Crick et al. estimated the dispersion radius of a merozoite group relative to the schizont mean radius to be 1.5 [64]. Taking that value to be *R/r* and using the hematocrit and schizontemia from our invasion assays, 2% and 0.7% respectively, we estimated a fraction accessible of less than 0.1%. However, in static culture, there is sedimentation of red blood cells, so the effective hematocrit could be much higher than 2%. Even assuming a hematocrit of 100% (keeping the same values for *R/r* and schizontemia), the fraction accessible increases to only 2.3%. Note that the expected volume fraction accessible is sensitive to *R/r* – increasing it from 1.5 to 2.5 (at 100% hematocrit) increases the volume fraction accessible to 10.4%.

Supplemental Table 1. Parameters of the within-host compartmental model.

| Symbol | Description | Value | Reference |
| --- | --- | --- | --- |
| Biological Parameters | | | |
| $\omega$ | Rate of hematopoiesis | 0.25 trillion  red blood cells (RBC) / day | Rate chosen to maintain 20-30 trillion circulating RBCs [53] |
| $f$ | Fraction of RBCs susceptible to invasion | 0.1, 0.25, 0.5 | – |
| $p$ | Probability of invasion given contact with susceptible RBC | 0.83, 0.33, 0.17 | For a fixed PMR, value determined by $f$ |
| $\tau$ | Duration needed for invasion, given contact has occurred | 5 min | [65] |
| $\beta$ | Rate of invasion per contact with susceptible RBC | 250, 100, 50 day^-1^ | Derived as $p/\tau$ |
| $\mu$ | Death rate for uninfected RBCs | 1/110 day^-1^ | [54] |
| $\mu_{I}$ | Death rate for infected RBCs | 1/2 day^-1^ | [59,60] |
| $\mu_{Z}$ | Death rate for merozoites | 1/5 min^-1^ | [65] |
| $m$ | Number of merozoites released per schizont | 15 | [60] |
| Structural Parameters | | | |
| $n$ | Number of successive compartments for susceptible and for resistant RBCs | 25 | – |
| $n_{I}$ | Number of successive compartments for infected RBCs | 25 | – |
